# Systematic review of barriers to and enablers of tuberculosis diagnosis, notification, and intervention for designing customised intervention package to minimise ‘missing millions’ in tribal communities of India

**DOI:** 10.7189/jogh.15.04303

**Published:** 2025-11-14

**Authors:** Ashish Satav, Dhananjay Raje, Vibhawari Dani, Radha Munje, Shraddha Kumbhare, Sanjay Zodpey, Manasi Shelgaonkar, Genevie Fernandes, Hilary Pinnock, Helen R Stagg, Harish Nair

**Affiliations:** 1 MAHAN Trust, Dharni, Amaravati, Maharashtra, India; 2 Department of Pulmonary, IGGMCH, Nagpur, Maharashtra, India; 3 Public Health Foundation of India, New Delhi, India; 4 International Primary Care Respiratory Group (IPCRG), Edinburgh, UK; 5 Primary Health Care Respiratory Medicine, University of Edinburgh, Edinburgh, UK; 6 Department of Infectious Diseases, London School of Hygiene & Tropical Medicine, London, UK; 7 Paediatrics Infectious Diseases and Global Health, University of Edinburgh, Edinburgh, UK

## Abstract

**Background:**

Tribal communities in India experience a very high burden of tuberculosis (TB), estimated at 7030 per million. The diagnosis and notification gaps are substantial, partly due to the geographical remoteness of these populations. Within an overarching study to design an intervention for finding the ‘missing millions’ among tribal communities, we conducted a systematic review to identify the barriers and enablers of tuberculosis diagnosis and notification, with the aim of developing a contextually relevant intervention.

**Methods:**

We searched PubMed, Embase and Web of Science using terms related to TB, diagnosis, notification, barriers, enablers, and interventions. Studies from lower- and lower-middle-income countries (LICs and LMICs) published between 2000–2023 were included. Qualitative and quantitative studies were assessed using the Critical Appraisal Skills Programme tool and Newcastle Ottawa scale, respectively. Narrative and thematic analyses were performed, applying the socio-ecological model (SEM) to categorise barriers and enablers of diagnosis and notification, and the consolidated framework for implementation research (CFIR) to assess intervention implementation.

**Results:**

Thirty-four eligible studies from 15 LICs and LMICs were included in the review. At community level, limited knowledge, illiteracy, stigma, geographical inaccessibility, and financial constraints were key barriers of diagnosis. At health system level, active case finding was the major intervention; however, inadequate diagnostic facilities, shortage of trained staff, insufficient incentives, weak counselling, and inadequate budget were the major barriers. Reported enablers were: increasing awareness about TB in the community to reduce stigma, encouragement from family members and TB survivors, mobilising human resources, regular capacity-building and monetary incentives to health workers.

**Conclusions:**

This systematic review identified barriers and enablers at multiple levels of the SEM and CFIR frameworks. To addressed the interconnected challenges, multifaceted and context-specific strategies are essential. Approaches that combine community engagement along with health system strengthening are essential for reducing the diagnosis and notification gaps among tribal populations.

**Registration:**

PROSPERO: CRD42023439841.

Tuberculosis (TB) is preventable and curable disease, but still kills more people globally per year than any other single infectious agent [1]. It was the leading cause of mortality with an estimated 1.25 million deaths in 2023. Worldwide, 8.2 million people were reported as newly diagnosed with TB in 2023, up from 7.5 million in 2022 and 7.1 million in 2019, of which 2.7 million were undiagnosed or not notified [2]. This notification gap is a key challenge to TB control programmes. In 2015, the World Health Organization (WHO) called for global concerted efforts to bridge the gap of the ‘missing millions’ as a part of their 2035 ‘End TB’ targets [3].

India had an estimated TB incidence rate of 196 / 100 000 (2.8 million individuals with TB) in 2022, a fifth of whom were not diagnosed/notified [4]. Amongst tribal populations, a previous systematic review and meta-analysis has revealed a pooled prevalence estimate of 7030 per million [5,6]. Under-diagnosis and delayed/missed notification in the country are unevenly spread and are more so in tribal populations. These populations often face systemic barriers to healthcare access, including geographical remoteness, limited transportation options, negligence from healthcare delivery system, absenteeism of doctors, cultural and linguistic differences and superstitions that leads to mistrust in the healthcare system. This eventually contributes to the high incidence, prevalence and mortality in such populations. In response to WHO’s ‘End TB’ effort, India has committed to eliminate TB through initiatives like the National Strategic Plan and Nikshay Platform. The elimination goal targets reducing TB incidence to 44 cases per 100 000 population and mortality to three per 100 000 by 2025 [7,8]. Achieving these targets needs to address case detection and notification gaps, especially in tribal populations.

Our research study primarily aims to design an intervention to address the diagnostic and notification gap in TB. To support this, we undertook a systematic review to examine the barriers and enablers influencing TB diagnosis and notification within the community, as well as those affecting the implementation of relevant interventions by health system, with a focus on low-income (LIC) and lower middle-income (LMIC) countries.

## METHODS

### Data sources and search terms

Three online bibliographic databases – PubMed, Embase, and Web of Science – were searched during September–October 2023. The search strategy included terms like tuberculosis, barriers, enablers, diagnosis and notification (Table S1 in the **Online Supplementary Document**). The syntax was framed according to the requirements of the database. The resultant was RIS format files from each database, which were extracted in COVIDENCE (Covidence systematic review software, Veritas Health Innovation, Melbourne, Australia) tool. The search was confined to titles, abstracts, and keywords. We restricted our search to studies published from the year 2000 due to the likely implications of the introduction of the WHO’s Directly Observed Treatment, Short Course (DOTS) framework, Stop TB and End TB strategies in the countries of our interest [9]. The reference lists of relevant systematic reviews were also screened.

### Study screening and inclusion exclusion criteria

Search lists were imported into COVIDENCE and duplicates were removed. Two reviewers (MS and SK) independently screened the titles and abstracts of articles to identify studies that met the inclusion/exclusion criteria of our two objectives *i.e*. barriers to and enablers of:

a) TB diagnosis and notification and

b) implementing interventions to overcome the barriers and enablers.

Any discrepancies between the two reviewers were addressed and resolved in consultation with a third reviewer (DR). Duplicate full text screening followed a similar process. The inclusion exclusion criteria were guided by PEO framework [10], defining the population, exposure and outcome to ensure systematic selection and evaluation of relevant studies (Table S2 in the **Online Supplementary Document)**. We included studies among current and ex-TB patients (all age groups, either gender) residing in rural or tribal settings of LICs and LMICs. Countries were classified according to their economic status at the time the study was undertaken, as per the World Bank classification [11]. Exposures were barriers of TB diagnosis, notification and intervention, while outcomes were TB diagnosis and notification. Studies on patients from higher income countries, patients with co-morbidities such as HIV, cancer, migrants were excluded. A total of 34 studies met the selection criteria and were included in the review.

### Data extraction

A data extraction template was created in COVIDENCE to capture essential details including: study setting, study type, non-healthcare and healthcare related barriers to and enablers of TB diagnosis and notification, and interventions. The data extracted by the two reviewers were resolved for discrepancies in discussion with DR. The overall inter-rater reliability was obtained through COVIDENCE. Mostly ‘positive’ results were extracted; while only a few studies reported ‘negative’ and ‘neutral’ results, which were also included. For instance, in quantitative studies, factors indicating barrier or enabler with high odds ratio (crude/adjusted) along with statistical significance were extracted, while for qualitative studies, the relevant factors from the thematic synthesis were reported.

### Quality assessment

We used the (CASP) checklist [12] for the assessment of the qualitative studies and the Newcastle-Ottawa scale for assessing the quality of quantitative and mixed methods studies [13]. During assessment, the criteria like appropriateness of qualitative methodology, the research design and its suitability to address the aims of the research, data collection and robustness of analysis, and the value of research in the present context were mainly weighted. In the quantitative assessment, factors like representativeness of the sample, response rate, statistical methodology and outcome assessment were given due weightage.

### Data analysis

Data on barriers and enablers from healthcare consumer’s (patient’s) perspective were mapped onto levels of the socio-ecological model (SEM) [14] (individual, interpersonal, organisational, community, and policy) and analysed thematically. Articles describing interventions and their barriers and enablers from healthcare system perspective were mapped according to the consolidated framework for implementation research (CFIR) framework [15] across five broad domains: individual characteristics, intervention characteristics, inner setting, outer setting and the implementation process and analysed thematically. The two frameworks were conceptually linked to elucidate the mutual influence of domains on each other.

### Registration

The protocol of this review was registered with the International Prospective Register of Systematic Reviews (PROSPERO) with registration number CRD42023439841.

## RESULTS

**Figure 1:**
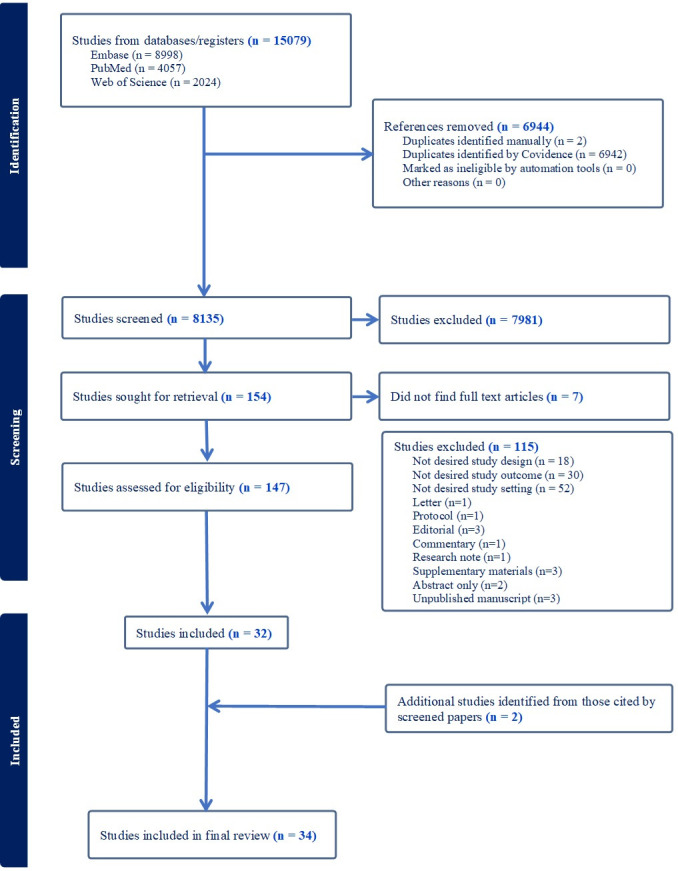
Prisma flow showing the process of article selection.

Our search yielded 8135 studies after duplicates were removed (**Figure 1**). After title, abstract and full-text screening, 32 studies were found suitable for inclusion. Two more studies were found from systematic reviews identified by our searches, resulting in 34 studies. The overall inter-rater reliability across title, abstract and full-text screening was 0.77, which was classified as ‘substantial’. The included studies and their characteristics are detailed in **Table 1**.

**Table 1:** Description of quantitative and qualitative studies included in the review

First author and year	Country name (classification)	Study setting	Study design	Study period	Population description	Sample size	Data collection method	Factors studied
Berhane Alema H, 2019 [16]	Ethiopia (LIC)	Institution based	CS	Nov 2015–Jan 2016	New PTB cases ≥15 years old	422	Structured questionnaire through interviewing	Barriers to TB diagnosis
Mihret AM, 2017 [17]	Ethiopia (LIC)	Institution based	CS	Apr 2013–Jul 2013	All newly diagnosed PTB patients ≥15 years old	605	Structured face-to-face interviewer-administered questionnaire	Barriers to TB diagnosis
Belkina TV, 2014 [18]	Uzbekistan (LMIC)	Institution based	CS	Aug 2013–Jan 2014	All newly diagnosed TB patients ≥15 years old	600	Structured questionnaire	Barriers to TB diagnosis
Selamsew Bogale E, 2017 [19]	Ethiopia (LIC)	Institution based	CS	Feb 2016–May 2016	Smear positive PTB cases >18 years old	311	Structured questionnaire through interviewing and record review	Barriers to TB diagnosis
Buregyeya E, 2014 [20]	Uganda (LIC)	Institution based	CS	Dec 2012–Mar 2013	Adult PTB patients within three months of initiating treatment	229	Structured questionnaire	Barriers to TB diagnosis
Cambanis A, 2007 [21]	Cameroon (LMIC)	Institution based	CS	2006	TB patients within all age groups	243	Structured questionnaire	Barriers to TB diagnosis
Choudhari MJN 2012, [22]	Nepal (LMIC)	Institution based	CS	Sep 2010–Jan 2011	PTB cases registered for DOTS	215	Pre-tested semi structured questionnaire	Barriers to TB diagnosis
Demissie M, 2002 [23]	Ethiopia (LIC)	Institution based	CS	Aug 1998–Dec 1998	Newly diagnosed PTB patients	700	Structured questionnaire through interviewing	Barriers to TB diagnosis
Terefe GF, 2018 [24]	Ethiopia (LIC)	Institution based	CS	May 2016–Sep 2016	TB patients of all age groups	398	Interview using a structured questionnaire	Barriers to TB diagnosis
Gebreegziabher SB, 2016 [25]	Ethiopia (LIC)	Institution based	CS	Oct 2013–Oct 2014	New PTB cases ≥15 years old	706	Pre-tested semi-structured questionnaire.	Barriers to TB diagnosis
Abdi AG, 2010 [26]	Ethiopia (LIC)	Both institution & community based	QR	Jun 2007–Sep 2007	1) Pastoralist TB patients; 2) Government officials	19	Informal interview techniques using an interview guide	Barriers to TB diagnosis
Islam MZ, 2020 [27]	Bangladesh (LMIC)	Institution based	RC	Jan 2018–Jun 2018	TB patients enrolled at DOTS centres	240	1) Face-to-face interview; 2) Medical records review using a semi-structured questionnaire	Barriers to TB diagnosis
Maamari F, 2008 [28]	Syrian Arab Republic (LIC)	Institution based	CS	3 Sep	New smear positive PTB patients ≥ 15 years old	800	Structured and pre-tested questionnaire	Barriers to TB diagnosis
Mbuthia GW, 2018 [29]	Kenya (LMIC)	Institution based	QR	Dec 2015–Mar 2016	TB patients in the intensive phase of treatment	61	1) Focus group discussions; 2) In- depth interviews	Barriers to TB diagnosis
Winters M, 2019 [30]	Uganda (LIC)	Institution based	CS	Sep 2015–Feb 2016	New smear positive PTB patients (adults)	134	Structured interviewer-administered questionnaire	Barriers to TB diagnosis
Osei E, 2015 [31]	Ghana (LMIC)	Institution based	CS	Jun 2013–May 2014	All newly diagnosed TB patients ≥15 years old	73	Pre-coded closed ended questionnaire	Barriers to TB diagnosis
Olumuyiwa AA, 2020 [32]	Gambia (LIC)	Institution based	CS	Oct 2016–Mar 2017	New smear positive PTB patients ≥ 18 years old	216	Structured questionnaire	Barriers to TB diagnosis
Rajeswari R, 2002 [33]	India (LMIC)	Institution based	CS	April 1997–Nov 1998	All newly diagnosed TB patients ≥15 years old	531	Structured questionnaire	Barriers to TB diagnosis
Zegeye MB, 2019 [34]	Ethiopia (LIC)	Institution based	CS	17 Dec	All TB patients who were taking anti-TB treatment	162	Structured questionnaire	Barriers to TB diagnosis
Verhagen LM, 2010 [35]	Tanzania (LMIC)	Institution based	QR	Sep 2008–Nov 2008	Patients with TB	30	Face to face interviews	Barriers to TB diagnosis
Yamasaki-Nakagawa M, 2001 [36]	Nepal (LMIC)	Community based	CS	Dec 1997–Jun 1999	Newly diagnosed PTB patients	408	1) Registration documents; 2) Face-to-face interviews using a questionnaire	Barriers to TB diagnosis
Yimer S, 2005 [37]	Ethiopia (LIC)	Institution based	CS	Sep 2003–Dec 2003	New smear positive PTB patients	384	Pre-tested semi-structured questionnaire	Barriers to TB diagnosis
Aye KW, 2010 [38]	Tajikistan (LMIC)	Both institution & community based	QR	2006	1) Community members; 2) Health care providers; 3) TB patients	97	Focus group discussions	Barriers and enablers to TB diagnosis
Babu Marahatta S, 2020 [39]	Nepal (LMIC)	Both Institution & community based	QR	2015	1) Community participants; 2) Government health service providers; 3) Private health service providers; 4) Traditional health service providers; 5) TB patients; 6) TB suspected patients	69	1) In-depth interviews; 2) Focus group discussions; 3) Semi-structured Interviews	Barriers and enablers to TB diagnosis
Msoka EF, 2021 [40]	Uganda, Kenya and Tanzania (LIC; LMIC)	Community based	QR	2016–2018	1) TB patients and survivors, caregivers; 2) General members of the community served by the healthcare facility; 3) Healthcare practitioners; 4) Local government authorities; 5) Policy/decision makers	712	1) In-depth interviews; 2) Key informant interviews; 3) Focus group discussions	Barriers and enablers to TB diagnosis
Teo AKJ, 2020 [41]	Cambodia (LMIC)	Institution based	MM	Feb 2019–Sep 2019	People with TB aged 18 above old	721	1) Face to face interviews; 2) In- depth interviews	Barriers and enablers to TB diagnosis
Mitano F, 2018 [42]	Mozambique (LIC)	Institution based	QR	May 2014–Aug 2014	1) Health professionals; 2) Physicians, technicians; 3) Nursing professionals	15	Interviews	Barriers to TB diagnosis and enablers to TB notification
Bhardwaj A, 2023 [43]	India (LMIC)	Institution based	MM	Jan 2019–July 2019	Private practitioners of the district	71	Semi-structured questionnaire-based interviews	Barriers and enablers to TB notification
Der JB, 2022 [44]	Ghana (LMIC)	Institution based	QR	May 2018–Jan 2019	Health care workers	12	1) Structured clinic observations; 2) In-depth interviews	Barriers and enablers to TB notification and intervention to improve TB diagnosis
Berhane Megerssa Ereso SAY, 2020 [45]	Ethiopia (LIC)	Institution based	QR	Aug 2016–Jan 2017	1) TB treatment providers; 2) Programme managers; 3) TB patient; 4) Health care workers	60	1) In-depth interviews; 2) Facility observations	Barriers to TB intervention
Korobitsyn A, 2013 [46]	Tajikistan (LMIC)	Institution based	QR	-			Random selection of primary documents	Barriers to TB intervention, enablers of notification
Shamanewadi AN, 2020 [47]	India (LMIC)	Institution based	QR	18 Jul	1) Health care providers; 2) Presumptive TB patients	25	In-depth interviews	Barriers and enablers to TB intervention
Legesse Tesfaye YKL, 2020 [48]	Ethiopia (LIC)	Both institution & community based	QR	Mar 2019–Apr 2019	1) TB coordinator; 2) Health Extension Workers; 3) Index TB patients; 4) Household contacts of active TB patients	16	1) In-depth interviews; 2) Interviews using a semi-structured guide	Barriers to TB intervention, enablers of notification
Yassin MA, 2013 [49]	Ethiopia (LIC)	Community based	MM	Oct 2010–Dec 2011	1) Community Members; 2) Health care providers	49 857	1) In-depth interviews; 2) Focus group discussions; 3) Retrospective surveillance data	Interventions to improve TB notification

### Characteristics of eligible studies

Out of 34 studies, 19 (55.9%) were quantitative, 12 (35.3%) qualitative and three (8.8%) were mixed-methods. The studies spanned 15 countries, eight from the African continent and seven from Asia. Twenty-seven studies (79.4%) described barriers and enablers of TB diagnosis [16-42] and four (11.8%) discussed barriers and enablers of TB notification [42-44,46]. Five (14.7%) studies assessed barriers and enablers of interventions at the community level; three (60.0%) from Ethiopia [45,48,49] and one each (20.0%) from Ghana [44] and India [47].

### Quality assessment

We performed the quality assessment of studies (Table S3, Panels A–B in the **Online Supplementary Document)**. On CASP appraisal, we observed that all qualitative studies (12) clearly defined their aims and study designs, and 10 (83%) had appropriate descriptions of their methodology. Nine (75%) studies properly collected data to address their research question and 11 (92%) clearly described their findings. Among the quantitative and mixed methods studies, all articles (n = 22) used a representative sample. The sample size justification was satisfactory with power calculations in 15 (68%) articles. The response rate was satisfactory in nine (41%) studies. The validated tools were used in nine (41%) studies, while 12 (55%) studies used non-validated tools, but were described appropriately. In all the studies, confounding was adjusted through multivariable or subgroup analysis. There were 18 (82%) studies with self-reported outcomes, while only four (19%) had independent blind assessment. Appropriate statistical evaluations were performed in 21 (95%) articles.

**Figure 2:**
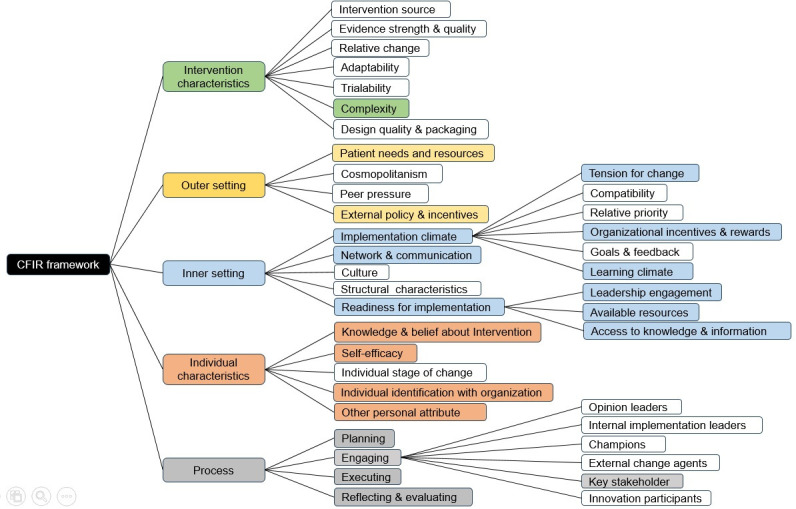
CFIR framework depicting relevant codes and subcodes identified from the study findings. CFIR – Consolidated Framework for Implementation Research.

The remainder of the results are presented as follows. First, barriers to, and then enablers of TB diagnosis from the quantitative and qualitative studies (**Table 2**), followed by barriers to, and then enablers of TB notification. Later part presents the thematic synthesis of the barriers/enablers of diagnosis and notification according to the SEM (Table S4 in the **Online Supplementary Document**), followed by the interventions (Table S5 in the **Online Supplementary Document)**, and then the synthesis of barriers and enablers of implementing interventions according to the CFIR framework (**Figure 2**; Table S6 in the **Online Supplementary Document**).

**Table 2:** Barriers and enablers of tuberculosis diagnosis classified according to the socio-ecological model

Socio-ecological model	Tuberculosis diagnosis: barriers	No. of studies	
		**Qualitative**	**Quantitative**
Individual	Old age (> 45 years)		10 [16,17,20,22,24,25,27,28,33]
	Illiteracy		11 [17,20-24,27,28,30,33]
	Lack of awareness/ knowledge of TB	7 [26,29,35,38-41]	10 [17,21,22,25,27,28,33,34]
	Patients attitude towards health care seeking	2 [29,38]	-
	Traditional medicines/ Traditional Faith Healer	4 [26,29,35,39]	6 [16,17,21,22,34,36]
	Self-medication	3 [29,40,41]	5 [16,18,21,22,25]
	Treatment from private health facility, multiple consultations	3 [29,35,41]	5 [18,19,31,32,37]
	Work and family commitments	2 [40,41]	-
	Loss of daily wage income		5 [16,17,22,24,25]
	Poverty/ financial constraint	3 [35,38,39]	8 [16,20-22,27,28,33,34]
	Occupation / working pattern	1 [40]	8 [16,17,22-25,28,30]
Interpersonal	Stigma associated with TB/ cultural beliefs	8 [26,29,35,38-42]	6 [16,17,21,25,28,31]
Organisational	Inadequate infrastructure	5 [26,29,38,40,42]	-
	Inadequate human resources	2 [26,39]	-
	Inadequate training of staffs	3 [38,39,42]	-
	Distance to health facility	6 [29,35,39-42]	10 [16,20-24,28,33,37]
	Lack of public transportation (accessibility)	4 [26,39,41,42]	-
	Symptomatic treatment by the health care providers	1 [35]	-
**Community**	-	-	-
Public policy	Inadequate finance/ funds for organisation	4 [26,29,38,39]	-
	Indirect cost/ Out of Pocket cost	4 [26,29,38,40]	-
	**Tuberculosis diagnosis: enablers**		
Individual	Fear of financial impact	1 [41]	-
	Knowledge and awareness about TB	2 [38,41]	-
	Fear of infecting others	1 [41]	-
Interpersonal	Encouragement from family members, social support	1 [41]	-
Organisational	Mobilisation of human resources	1 [39]	-
	Upgrade infrastructure	1 [39]	-
	Organisational collaboration	1 [39]	-
	Health education by health staff	1 [40]	-
Community	Knowledge and awareness about TB	2 [38,41]	-
Public policy	-		

### Socioecological model: barriers to tuberculosis diagnosis

#### Individual

The barriers to early TB diagnosis encompass a range of factors affecting different segments of the population. Older age (≥ 45 years) was consistently linked to longer delays in diagnosis; this was observed in nine studies [16,17,20,22,24,25,27,28,33]. However, in a study by Demissie et al. (2002), the authors reported that older age is not a barrier in diagnosis because of the variations in cultural attitudes toward elderly healthcare [23]. Illiteracy amplifies patient delay, with 10 studies highlighting the impact of educational levels on healthcare-seeking behaviour [17,20-24,27,28,30,33]. On the contrary, Gebreegzi et al. (2016) observed that illiteracy among patients led to early diagnosis of TB [25]. Lack of awareness and knowledge about TB emerged as an important barrier, as evidenced by 15 studies [17,21,22,25-29,33-35,38-41]. While Alema et al. (2019) and Fuge et al. (2018) observed that poor knowledge about TB led to early diagnosis [16,24]. Patients rely more heavily on traditional community networks and family support systems that facilitate rapid healthcare-seeking when symptoms appear. Patients showed reluctance to seek care from public/private healthcare facilities, [29,38] preferring traditional healers due to their belief systems or the stigma associated with a TB diagnosis [16,17,21,22,26,29,34-36,39]. Work and family commitments hindered timely medical attention, [40,41] exacerbated by concerns over the loss of daily income [16,17,22,24,25]. Financial constraints, including the cost of travel, and treatment, posed important obstacles to seeking care for TB symptoms, as highlighted in 11 studies [16,20-22,27,28,33-35,38,39]. Occupation and working patterns, such as farming or migration, presented challenges in accessing healthcare services promptly [16,22-24,28,30,40]. Asres et al. (2017) and Gebreegzi et al. (2016) observed that daily labourers and farmers have lesser barrier towards diagnosis of TB [17,25]. Targeted public health programmes prioritise outreach to agricultural and labour-intensive communities, recognising these populations as high-risk groups for TB transmission.

#### Interpersonal

The stigma associated with TB and cultural beliefs were important barriers to early detection, as shown by 14 quantitative and qualitative studies. Fear of exclusion from the community and concerns about being HIV-positive contributed to individuals avoiding getting a TB diagnosis [16,17,21,25,26,28,29,35,38-42]. The universal presence of stigma-related barriers indicates that addressing social and cultural perceptions of TB remains a critical component of any comprehensive strategy to improve early diagnosis rates.

#### Organisational

At the organisational level, patient reported inadequate infrastructure [26,29,38,40,42] and insufficient human resources [26,39] emerged as the key barriers. Further, long distances to a health facility and lack of transportation were the major barriers to diagnosis [16,20-24,28,29,33,35,37,39-42]. However, Gebreegzi et al. (2016) reported that even more than two hours of travel time to hospital due to long distance was not a barrier in diagnosis [25]. Patients in this particular context have had greater health awareness that motivated them to overcome distance barriers. Factors such as poor road conditions, challenging geographical terrain, and seasonal extremes like heavy rainfall or high temperatures caused further issues. Early TB detection gets complicated by healthcare providers’ tendency to focus on symptomatic treatment rather than pursuing definitive TB was diagnosis, which delays proper care initiation [35].

#### Public policy

The indirect costs incurred by patients for travel, food, and accommodation presented challenges in accessing diagnostic services and continuing treatment after TB detection [26,29,38,40]. The economic strain may force patients to choose between seeking healthcare and meeting basic family needs, ultimately perpetuating the cycle of delayed diagnosis and continued TB transmission within communities.

### Socioecological model: enablers of tuberculosis diagnosis

There were seven studies evaluating the factors enabling TB diagnosis. They are described according to the socio-ecological model below:

#### Individual

Increasing knowledge and awareness about TB in the community emerged as a key enabler for early diagnosis [38,41]. Enhanced community understanding of TB symptoms, transmission patterns, and treatment availability empowers individuals to recognise potential signs of infection and seek timely medical attention. Teo et al. highlighted the fear of infecting others and the adverse effects of the condition on well-being, work, and livelihoods as reasons for seeking diagnosis [41]. Patients undergo TB testing by fear of infecting family members, friends, or community members, demonstrating how social responsibility can facilitate early healthcare-seeking behaviour.

#### Interpersonal

Encouragement from family members and other TB survivors served as an impetus for TB care-seeking in the study by Teo et al*.* [41]. Family support provides emotional reassurance in overcoming barriers to healthcare access, helping patients navigate the healthcare system and cope with TB-related stigma. The influence of TB survivors is particularly valuable.

#### Community

There were two studies highlighting the importance of knowledge and awareness about TB at the community level to reduce stigma associated with TB in the community [38,41]. Enhanced community education about TB's transmission mechanisms, curability, and prevention helps dispel myths and misconceptions that fuel discriminatory attitudes toward TB patients. This improved community awareness creates an environment where TB patients feel safer seeking diagnosis and treatment without fear of social stigma.

### Barriers and enablers of tuberculosis notification

There were four studies describing barriers to, and enablers of TB notification. Three studies discussed barriers to timely TB notification, particularly barriers within healthcare systems [42-44]. Poor documentation practices among healthcare staff at municipal hospitals, often attributed to laziness, lead to incomplete or inaccurate reporting of TB cases [44]. Additionally, staff shortages or high turnover rates lead to gaps in personnel, exacerbating the workload and impeding notification efforts [42,44]. The absence of modern technology for efficient data transmission further complicates the process [42]. Complex notification procedures, coupled with lack of coordination between public and private sectors, create additional challenges [43]. Insufficient training for healthcare professionals on TB notification protocols, coupled with a lack of incentives for reporting, undermines effective notification efforts [43]. Moreover, inadequate education on TB mandatory notification policies adds to the difficulty in ensuring timely reporting of cases [43].

Several measures were discussed as enablers to improve TB notification in three studies [42,43,46]. Strengthening the surveillance system is crucial, ensuring accurate and timely reporting of TB cases [46]. Establishing links with a broad spectrum of healthcare providers facilitates effective information exchange, enhancing the overall notification process [46]. Implementing corrective measures for early patient default helps address gaps in TB care continuity. Stronger enforcement of legislation regarding case notification reinforces the importance of reporting [46]. Promoting understanding among healthcare workers regarding the role of case reporting in ensuring that TB activities can be properly planned enhances compliance [42]. Furthermore, providing comprehensive training to healthcare professionals who are committed to TB work for long durations fosters expertise and continuity in TB care [42]. Group sensitisation sessions for healthcare providers aids in raising awareness and promotes a collective approach to TB notification efforts [43].

### The interventions

Out of the five intervention-related articles, four articles focussed on screening intervention (TB case finding) [44,45,47,48], while only one article by Yasin (2013) [49] developed and implemented an intervention package to address the barriers, which was presented through a comparative study (Table S5 in the **Online Supplementary Document**). These studies discussed barriers/enablers of implementing interventions, as faced by the individual and health-care system. The barriers/enablers were organised according to the Consolidated Framework for Implementation Research framework, along with the thematic synthesis (**Figure 2**; Table S6 in the **Online Supplementary Document)**.

### Consolidated Framework for Implementation Research (CFIR)

#### Intervention characteristics

In this domain, the specific code identified in the review was complexity. Mismanagement of equipment stocks by the staff and unavailability in urgent situations were reported as the causes of delayed diagnosis and inconvenience to patients, leading to complexity in implementing interventions. Health centres with limited or no diagnostic facilities rely on other, better equipped, health centres. Further, long distances affecting the transportation of samples from one centre to other, and financial constraints were stated as the reasons for delayed diagnosis [44,45]. Often, government healthcare workers have a defined target for sample collection, which may lead to underdiagnosis. Moreover, compromised sample quality leading to misdiagnosis or delays in diagnosis was also reported as a major concern [47].

To overcome these complexities, the provision of mobile phone airtime credits to healthcare workers, motorcycles for timely transport of samples, use of light-emitting-diodes-fluorescent microscopes (LED-FM) for laboratories, and messaging about TB support services through community gatherings were built into the intervention to enhance it [49].

#### Outer setting

One of the codes identified in this domain was patient needs and resources. Patient behaviour can hinder the healthcare system from timely diagnosis and treatment [44,47,48]. Major barriers included traditional beliefs and stigma in the community. Moreover, a lack of respect from community members towards healthcare workers was also noted to hamper intervention use [47].

Engagement of local stakeholders at various levels including village leaders, politicians and religious leaders were suggested as enhancers to intervention roll-out. Further, holding regular community meetings, campaigns to disseminate TB related information, and home visits by field staff were also reported as enablers [48,49].

The other code identified was external policies and incentives, which aims at external strategies to intensify the intervention. The collaboration with national TB control programmes and outside agencies for feedback, evaluation and support in improving the quality of TB control activities was suggested as a strong enabler [49].

#### Inner setting

In this domain, the codes identified were implementation climate, network communication and readiness for implementation. For the implementation climate, we observed the sub-codes: tension for change, organisational incentives and rewards and learning climate. For readiness for implementation, the sub-codes identified were: leadership engagement, available resources and access to knowledge and information. Stakeholders at the field level suggested the need for changes in the implementation process. Low monetary incentives demotivate healthcare workers, resulting in poor performance [47]. Further, a lack of training for field staff on how to collect samples or conduct active case finding (ACF) were identified as the key barriers under learning climate. Insufficient monitoring and supervision by higher health authorities affected leadership engagement [44,47,48]. Additionally, inappropriate design of health facilities, the lack of a clean water supply, electricity and rooms for TB treatment, poor levels of maintenance and excessive workloads placed on existing staff were the major barriers for implementation. Closing of training institutes – which then hampered access to knowledge and information- was also a major interventional barrier [45,48].

The appointment of supervisors at the district level for monitoring the implementation process acted as a facilitator. Further, the familiarity of healthcare workers with the workplace and community’s comfortableness with the field workers proved to be facilitators of implementation. Capacity building workshops for health workers and laboratory staff also acted as enhancers [49].

#### Individual characteristics

The codes identified in this domain were: knowledge and belief about the intervention, self-efficacy and other personal attributes. Limited knowledge about TB interventions, lack of understanding about case detection guidelines and procedures, and poor adherence were major barriers to implementation [44,48]. A further barrier was that the infectious nature of TB resulted in fear among healthcare workers and discouraged them from fully committing to the intervention [44,45]. Poor counselling skills also acted as barrier [47]. These barriers hamper healthcare worker self-efficacy, motivation and competency level.

However, the perceived usefulness of ACF among the field staff, and skills/knowledge enhancement during intervention roll-out among healthcare workers and laboratory technicians acted as facilitators to implementation [45,47].

#### Implementation process

The codes identified in this domain were: planning, executing, engaging and reflecting, and evaluating. Inappropriate resource mobilisation and inadequate budget allocation were identified as key barriers in the planning process [44,45,47,48]. Further, complicated procedures of referral, feedback and linkage of health facilities, and the ineffective management of field hours leading to the inability to interact with community were reported as causes of delays [48]. Poor documentation hindered the monitoring and evaluation of ACF in rural settings [44].

House-to-house field visits by healthcare workers for active case finding, collection of sputum samples, treatment initiation and patient monitoring, reporting of treatment outcomes and side effects, strict supervision by the field supervisors and improved communication facilities were the identified facilitators in this domain [49]. Engaging key stakeholders like political, community and religious leaders, as well as teachers and ex-TB patients and arranging workshops/meetings, community engagement activities to encourage participation would enable the implementation process. Routine data collection for surveillance, performance evaluation by independent experts and submission of reports to national and international agencies for feedback and further improvement were suggested as the key enablers of innovative interventions [49].

## DISCUSSION

**Figure 3:**
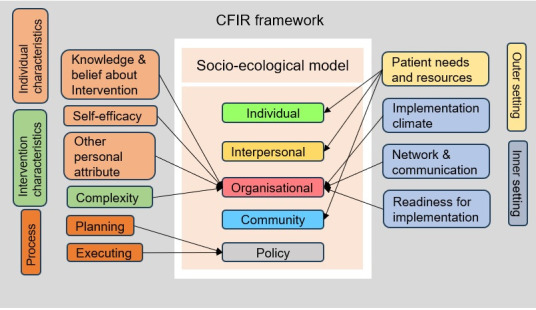
Mapping of CFIR domains onto SEM levels showing reciprocal influence. CFIR – consolidated framework for implementation research, SEM – socio-ecological model.

This systematic review included qualitative, quantitative and mixed methods studies to determine the barriers and enablers to TB diagnosis and notification in LICs and LMICs, as well as barriers and enablers of implementing the interventions. Patient perceptions were integrated into the socio-ecological model, while the healthcare insights were organised according to CFIR framework. The influence of domains from the two frameworks on each other was studied (**Figure 3**). The associations were referred to design interventions aimed at mitigating gaps in tuberculosis diagnosis and notification.

### Barriers and enablers of TB diagnosis

In the socio-ecological model, at individual level, lack of TB awareness and knowledge emerged as a prominent barrier to TB diagnosis across studies at individual level. This aligns with the findings from India, where low TB literacy has been associated with delayed care-seeking [50,51]. The review highlighted how illiteracy and poor socio-economic conditions compound this issue, particularly in rural and tribal settings, which resonates with the work from tribal areas of India [52]. These conditions underpin reliance on traditional faith healers, thereby impeding timely access to the formal healthcare system [40]. At community level, cultural beliefs surrounding TB and fear of social discrimination prevents individual from disclosing their health condition that hinders the way to timely diagnosis and treatment [39,44,53]. These socioeconomic model-based individual, interpersonal and community level barriers were associated with the sub-code Patient needs and resources of CFIR framework (**Figure 3**). Despite various intervention programmes in different LMICs for TB prevention and control, misconceptions about interventions remain prevalent adversely affecting individual’s healthcare seeking behaviour. This poses a challenge to the health system in understanding the patient’s needs and implementing timely interventions [52]. Addressing these barriers necessitates targeted health education and awareness campaigns. The complexity of intervention, a sub-code from CFIR framework, was a major barrier from healthcare provider’s perspective. In LMICs, primary healthcare delivery is hindered by inadequate infrastructure, insufficient training to staff, poor management of diagnostic equipment, consumables supplies and modern technology for efficient information transfer [44,45,48]. These are allied with the organisational domain from the socioecological model. Patient’s experiences due to organisational-level barriers often deter them from seeking treatment from health centres. Furthermore, the CFIR sub-codes Implementation climate, network, and communication and readiness for implementation were identified as key barriers to the intervention process. Low monitory benefits, lack of training for sample collection and TB case detection, inadequate monitoring and supervision by senior staff were the demotivating factors for effective implementation of interventions [44]. These systemic issues have been widely recognised in the Indian context as impediments to achieving TB control targets [54,55]. The Knowledge and belief about intervention, Self-efficacy and Personal attributes were also the prominent CFIR sub-codes affecting the Organizational level performance [44,48]. Quite often healthcare workers have poor counselling skills, self-confidence to convince suspected cases for investigations and treatment, [47] which could be because of partial knowledge about case detection and treatment procedures. These factors contribute to individual’s distrust in the organisation. Deploying a robust implementation Process with proper Planning, Stakeholder engagement and Execution are fundamental to mitigate these barriers. Adequate human resource with simple referral procedures, networking of health facilities for easy sample collection and analysis, suitable budget allocations to meet operational expenses and incentives to healthcare professionals are the key enhancers to overcome the organisational barrier [39,40,44]. Furthermore, the distance to healthcare facilities, lack of public transportation, poor road conditions, impede access to TB diagnosis and treatment [29,38,40,48]. The indirect cost borne by patients for commuting, accommodation and food further aggravates, requires attention during policy making.

The CFIR framework underscored health system challenges in implementing TB case finding programmes, although these have previously been found to be successful in different vulnerable groups in India [56,57]. The integration of CFIR and socio-economic model offers valuable insights for prioritising barriers when designing tailored interventions to improve TB diagnosis.

### Barriers and enablers to TB notification

WHO provides a global framework for TB notification, emphasising the importance of mandatory reporting of all TB diagnosed cases. The aim is to reduce TB incidence by 80% and TB deaths by 90% by 2030, with a focus on improving the notification system. Several LMICs are transitioning to digital platforms for TB notification, such as India’s Nikshay portal [58]. Despite robust notification system, the challenges such as poor documentation and lack of coordination between public and private sectors, data inaccuracies exist and are aggravated due to technology and infrastructure barriers [42-44]. Earlier, Yassin (2013) stated that in their intervention package, the supervisors supported the routine recording and reporting systems to enhance notification. The suggested enablers, including strengthening surveillance systems and promoting understanding among healthcare workers about the importance of case reporting, align with the ongoing efforts to improve India's Nikshay portal for TB notification.

### Customised intervention for a tribal setting of India – Melghat

The intervention package outlined below adopts a comprehensive multi-level strategy to enhance TB diagnosis and notification in Melghat, a tribal setting of central India referring to the interlinking of socio-ecological model and CFIR frameworks. Cross-level integration ensures that interventions across the socio-ecological spectrum work synergistically, with established feedback mechanisms linking community needs to policy responses and enabling continuous local adaptation. Cultural sensitivity remains paramount, requiring deep engagement with local communities as true partners in design and implementation, while respectfully integrating traditional healing practices with biomedical TB care.

Individual level interventions are:

• Awareness and Education: develop culturally adapted Behaviour change communication (BCC) and Information, Education, Communication (IEC) tools using local dialects and pictorial messages to overcome illiteracy.

• Symptom Recognition: use trained village health workers to conduct door-to-door awareness programmes on early identification of symptoms of TB.

• Economic Support: link patients to government nutrition and livelihood schemes (*e.g*. Nikshay Poshan Yojana), to reduce loss of daily wages and treatment discontinuation.

Interpersonal level interventions are:

• Family Engagement: involve family members in counselling sessions to reduce stigma and encourage adherence.

• Peer Support: form TB survivor groups who can serve as role models and motivators for early health-seeking behaviour.

Community level interventions are:

• Traditional Healer Integration: engage traditional faith healers/Accredited Social Health Activist (ASHA) to act as referral partners instead of barriers.

• Community Mobilisation: regular Gram Sabha (community meetings) discussions and health fairs to normalise TB dialogue, reduce stigma, and promote collective responsibility.

• Mobile Diagnostic Camps: periodic outreach in remote hamlets with sputum collection and digital chest X-rays to overcome distance barriers.

Organisational/Health System Level interventions are:

• Decentralised Diagnostics: establish sputum microscopy and RT-PCR (CBNAAT) services at block and Primary Health Centre levels; use solar-powered equipment for areas with poor electricity.

• Workforce Strengthening: train and incentivise ASHAs, Auxiliary Nurse Midwife (ANMs), and local tribal youth as TB champions for case detection and treatment support.

• Technology Use: strengthen use of Nikshay for real-time notification, supported by offline data entry for low-connectivity zones.

Policy and Structural Level interventions are:

• Transport Solutions: provide bicycles or motorcycles to health workers for sample transport.

• Cross-sectoral Collaboration: align with tribal welfare, forest, Integrated Child Development Schemes (ICDS) and education departments to integrate TB awareness in schools and livelihood programmes.

• Accountability and Incentives: introduce recognition and small financial incentives for frontline staff ensuring early diagnosis and complete notification.

Implementation Enablers (CFIR-linked) are:

• Leadership Engagement: district TB officers and community leaders jointly monitor implementation.

• Capacity Building: regular training and refresher workshops for health workers, lab staff, counsellors and local NGOs.

• Community Participation: ex-TB patients, gaon panchayat (traditional village council) teachers, traditional faith healers, sarpanch (village elected member), police patil (Government appointed) and religious leaders included in advisory committees.

• Monitoring an Evaluation:

o Process indicators: number of people screened (Sputum microscopy and X-Ray chest), diagnostic turnaround time, notification rates.

o Outcome indicators: treatment initiation, completion rates, stigma reduction.

o Participatory mechanisms: community scorecards and feedback loops to adapt interventions in real time.

Cultural Sensitivity for Melghat:

• Use Korku and Gondi language materials, folk songs, and street plays for communication.

• Respect tribal healing traditions while educating about biomedical treatment – encouraging a dual care pathway rather than confrontation.

• Gender-sensitive approaches: involve women’s self-help groups to address stigma and treatment adherence among women.

A cluster randomised control trial is under development to show the impact of the above interventions. We will ensure that village health workers will screen all suspected cases in the community and provide appropriate referrals to hospitals for the confirmation of diagnosis and initiation of treatment. They will also ensure follow-up of all patients till the completion of treatment. By weaving together individual, family, community, organisational, and policy interventions – designed with cultural humility and real-time feedback – the Melghat TB programme can significantly reduce diagnostic delays, improve notification, and contribute to closing the ‘missing millions’ gap in TB care.

On similar lines, this review will guide the development of tailored interventions among the rural / tribal populations in other LICs and LMICs across globe. A robust monitoring and evaluation framework can incorporate level-specific indicators, both process and outcome measures, and community-participatory evaluation mechanisms to track progress and guide improvements.

### Limitations

One of the limitations of this review was that the reference population was primarily from LICs or LMICs; however, developed nations are also facing TB related issues due to illegal migrations from neighbouring countries, and their issues were untouched in this review. Studies on ‘negative’ or ‘neutral’ results were few, which possibly could have altered the balance of evidence for each factor. Another limitation was the inclusion of studies published in English language only; however, the strength of the review is the wide coverage of LICs and LMICs, enabling generalisable findings on barriers and enablers of TB diagnosis through robust thematic synthesis. Furthermore, the dual-framework approach and their interlinkage are expected to facilitate the prioritisation of thrust areas for the development of tailored interventions. Unfortunately, in comparison to diagnosis, studies looking to improve TB notifications were limited. Furthermore, there were hardly any studies on interventions to overcome barriers of diagnosis and notification through proper study designs.

## CONCLUSIONS

This systematic review highlights the complex interplay of factors influencing TB diagnosis and notification and emphasises the need for a multifaceted approach to finding the missing millions in LICs/LMICs. Addressing the barriers through targeted intervention at various levels of socio-ecological framework and the CFIR can significantly enhance TB control efforts. Tailored strategies for education, community engagement, setting of mobile diagnostic units, strong local health workforce, organisational improvements and supportive policies are crucial for improving TB diagnosis and notification rates. Tribal-focused interventions is an urgent operational necessity to meet national level targets in these countries, ultimately leading to improved TB control indicators.

## Additional material


Online Supplementary Document
