# Supplementary material for: Systematic review of barriers to and enablers of tuberculosis diagnosis, notification, and intervention for designing customised intervention package to minimise ‘missing millions’ in tribal communities of India
Source: J Glob Health. 2025 Nov 14;15:04303. doi: 10.7189/jogh.15.04303 (PMC12616580; doi:10.7189/jogh.15.04303)
Supplement: Online Supplementary Document [file jogh-15-04303-s001.pdf]

Supplement to: Satav A, Raje D, Dani V, Munje R, Kumbhare S, Zodpey S, Shelgaonkar M, Fernandes G, Pinnock H, Stagg HR, Nair H. Systematic review of barriers to and enablers of tuberculosis diagnosis, notification, and intervention for designing customised intervention package to minimise 'missing millions' in tribal communities of India. J Glob Health. 2025;15:04303.

Table S1: Search strategy for study selection

**PubMed**	((Tuberculosis [MeSH] **OR** Tuberculosis [tw]) **AND** ((Barrier*[tw] **OR** Obstacle*[tw] **OR** "Hard to reach"[tw] **OR** "Patient Acceptance of Health Care"[Mesh]) **OR** (Enabler*[tw] **OR** Facilitator*[tw] **OR** Promoter*[tw] **OR** Supporter*[tw])) **AND** ((Notification[tw] **OR** Reporting[tw] **OR** "Case notification"[tw] **OR** "Healthcare seeking behavior"[tw] **OR** "Missing millions"[tw] **OR** Underreporting[tw] **OR** "Under-reporting"[tw] **OR** "Disease Notification"[MeSH]) **OR** (Diagnosis [tw] **OR** Finding[tw] **OR** Screening [tw] **OR** "Active case finding" [tw] **OR** "Detection"[tw] **OR** "Delayed diagnosis"[tw] **OR** "Diagnostic Testing"[tw] **OR** "Diagnostic delay"[tw] **OR** Tuberculosis/Diagnosis [MeSH])))
**EMBASE (Ovid)**	((exp Tuberculosis/ **OR** Tuberculosis.mp.) **AND** ((Barrier*.mp. **OR** Obstacle*.mp. **OR** "Hard to reach".mp. **OR** exp "Patient Acceptance of Health Care"/) **OR** (Enabler*.mp. **OR** Facilitator*.mp. **OR** Promoter*.mp. **OR** Supporter*.mp.)) **AND** ((Notification.mp. **OR** Reporting.mp. **OR** "Case notification".mp. **OR** "Healthcare seeking behavior".mp. **OR** "Missing millions".mp. **OR** Underreporting.mp. **OR** Under-reporting.mp. **OR** exp "Disease Notification"/) **OR** (Diagnosis.mp. **OR** Finding.mp. **OR** Screening.mp. **OR** "Active case finding".mp. **OR** Detection.mp. **OR** "Delayed diagnosis".mp. **OR** "Diagnostic Testing".mp. **OR** "Diagnostic delay".mp. **OR** exp Tuberculosis*/Diagnosis)))
**Web of Science**	(Tuberculosis **AND** ((Barrier* **OR** Obstacle* **OR** "Hard to reach" **OR** (Enabler* **OR** Facilitator* **OR** Promoter* **OR** Supporter*)) **AND** ((Notification **OR** Reporting **OR** "Case notification" **OR** "Healthcare seeking behavior" **OR** "Missing millions" **OR** Underreporting **OR** Under-reporting **OR** "Disease Notification") **OR** (Diagnosis **OR** Finding **OR** Screening **OR** "Active case finding" **OR** Detection **OR** "Delayed diagnosis" **OR** "Diagnostic Testing" **OR** "Diagnostic delay"))

Table S2: Inclusion and exclusion criteria as per PEO framework- Barriers/enablers to TB diagnosis, notification and intervention

Parameter	Inclusion criteria	Exclusion criteria
**P**opulation	Current and ex-TB patients of all age groups of either gender residing in rural or tribal settings of lower- and lower-middle income countries.	Studies on patients from higher income countries, patients with co-morbidities such as HIV, cancer, migrants.
**E**xposure	Barriers and enablers of TB diagnosis, notification and intervention	
**O**utcomes	TB diagnosis and notification	

Table S3-A: Assessment of qualitative studies using CASP scale

Questions	Aye KW 2010 [38]	Der JB 2022 [45]	Berhane Megerssa Ereso SAY 2020 [46]	Abdi AG 2010 [26]	Korobitsyn A 2013 [43]	Babu Marahatta S 2020 [39]	Mbuthia GW 2018 [29]	Mitano F 2018 [42]	Msoka EF 2021 [40]	Shamanewadi AN 2020 [49]	Legesse Tesfaye YKL 2020 [47]	Verhagen LM 2010 [35]
Was there a clear statement of the aims of the research?	Yes	Yes	Yes	Yes	Yes	Yes	Yes	Yes	Yes	Yes	Yes	Yes
Was a qualitative methodology appropriate?	Yes	CS	Yes	Yes	CS	Yes	Yes	Yes	Yes	Yes	Yes	Yes
Was the research design appropriate to address the aims of the research?	Yes	Yes	Yes	Yes	Yes	Yes	Yes	Yes	Yes	Yes	Yes	Yes
Was the recruitment strategy appropriate to the aims of the research?	Yes	Yes	Yes	Yes	CS	Yes	Yes	Yes	Yes	Yes	CS	Yes
Was the data collected in a way that addressed the research issue?	Yes	Yes	Yes	CS	CS	Yes	Yes	Yes	Yes	Yes	CS	Yes
Has the relationship between researcher and participants been adequately considered?	No	Yes	Yes	No	CS	Yes	Yes	Yes	No	No	No	Yes
Have ethical issues been taken into consideration?	CS	CS	Yes	Yes	CS	Yes	Yes	Yes	Yes	Yes	Yes	CS
Was the data analysis sufficiently rigorous?	No	Yes	CS	CS	Yes	Yes	Yes	Yes	Yes	Yes	CS	CS
Was there a clear statement of findings?	Yes	Yes	Yes	No	Yes	Yes	Yes	Yes	Yes	Yes	Yes	Yes

How valuable was the research?	Yes	Yes	CS	CS	CS	Yes	Yes	Yes	Yes	CS	CS	CS

CS: Can't say

Table S3-B: Assessment of quantitative studies using Newcastle-Ottawa scale*

First author Year [Reference]	Representativeness of the sample	Sample size	Non-respondents	Ascertainment of the exposure	Comparability	Assessment of the outcome	Statistical test
Berhane Alema H 2019 [16]	1	1	0	1	1	2	1
Mihret AM 2017 [17]	1	1	0	1	1	2	1
Belkina TV 2014 [18]	1	1	1	2	1	1	1
Selamsew Bogale E 2017 [19]	1	1	0	2	1	1	1
Buregyeya E 2014 [20]	1	0	1	2	1	1	1
Cambanis A 2007 [21]	1	0	0	1	2	1	1
Choudhari MJN 2012 [22]	1	0	0	2	1	1	1
Demissie M 2002 [23]	1	1	0	1	1	2	1
Terefe GF 2018 [24]	1	1	1	1	1	1	1
Gebreegziabher SB 2016 [25]	1	1	1	2	1	1	1
Islam MZ 2020 [27]	1	1	0	1	1	2	1
Maamari F 2008 [28]	1	1	0	2	1	1	1
Winters M 2019 [30]	1	0	0	0	1	1	1
Osei E 2015 [31]	1	0	1	1	1	1	1
Olumuyiwa 2020 [32]	1	0	0	1	1	1	1
Rajeswari R 2002 [33]	1	0	1	1	2	1	1
Zegeye MB 2019 [34]	1	1	1	2	1	1	1
Teo AKJ 2020 [41]	1	1	1	2	1	2	1
Yamasaki-Nakagawa M 2001 [36]	1	1	0	0	1	1	1
Yimer S 2005 [37]	1	1	0	2	1	1	1
Bhardwaj A 2023 [44]	1	1	1	1	2	1	0
Yassin MA 2013 [48]	1	1	0	1	2	1	1

**For cross-sectional studies.*

Table S4: Thematic synthesis of barriers and enablers of tuberculosis diagnosis from qualitative studies, as per the socio-ecological model

Socio-ecological model - Code	Theme: Barriers to TB diagnosis	Quotes
Individual	Lack of awareness about TB, poor knowledge about symptoms as well as treatment of TB, belief that TB is punishment against sinful acts.	*"…TB is transmitted by doing heavy jobs like farming and engaging in sexual intercourse with girls and smoking" [TB Patient, 1 TZ]. Msoka EF 2021 [40]*<br><br>*". . . You know most severe disease comes as a result of curses and witchcraft; it is only Chepsakitian . . .who can cure you of such." (2nd female FGD). Mbuthia GW 2018 [29]*
Individual	People with casual attitude towards treatment of TB, priority towards alternative, cost-effective medication	*". . .that time it was not very bad. As a man, you don't fall sick today, and tomorrow you are in the hospital. We go to the hospital when it is very severe." (IDI Male 32 years) Mbuthia GW 2018 [29]*<br><br>*". . .but with us men when you fall sick with a minor problem like coughing we don't act fast. We usually persevere first because you have other things to do to earn a living." (IDI Male 37 years). Mbuthia GW 2018 [29]*
Individual	Traditional medicines, faith on traditional healers, convenience of treatment.	*"I used the herbal drugs for a period of one year. You know they are of different types from different trees. So what we do we try this one when it fails you try the other." (IDI Female 39 years) Mbuthia GW 2018 [29]*<br><br>*"I tried local herbs, I avoided sex, I tried religious remedies in many occasion and I didn't seek treatment until my situation reached to a point that I couldn't milk camels for my children" (Nomadic pastoralist, Jigjiga DOT center). Abdi AG 2010 [26]*
Individual	Self-medication from pharmacy and ignorance towards symptoms	*"In my case, I was sick for almost two years. I tried many things I bought medicine in the shops, I went to the local medicine man who gave me some herbs, but this did not help. I also went to the dispensary many times, but they treated me for other things. I had a severe cough for two months after which I came here, and my sputum was tested, and they found TB." (2nd female FGD) Mbuthia GW 2018 [29]*<br><br>*"I never went to hospital. When I was [had] cough, I was buying [bought] medicines from pharmacy." (IDI26, 68yo, male, rural, long delay) Teo AKJ 2020 [41]*
Individual	Treatment from private health facility,	*"I had started coughing long before, but I only bought medicines from the shops. I did*

	multiple consultations from private healthcare	*not improve, and when it got worse I went to a private clinic in January where I was told that it was Pneumonia. . . But my illness got worse . . . I therefore decided to seek treatment in a bigger hospital and came to. . . hospital. . . and I was found to have TB." (IDI Male 27 years). Mbuthia GW 2018 [29]*<br><br>*"We don't have enough money because when I got sick, I spent 2 thousand to 3 thousand dollars [on multiple consultations and treatments]. I couldn't go to work. I stayed at home." (IDI, 32yo, female, rural, short delay) Teo AKJ 2020 [41]*
Individual	Type of occupation / working pattern become barriers to early diagnosis	*'…The young men working in mining areas because we are at a very high risk of contracting TB but when we fall sick, our bosses do not give us anything…' [Community member, 2 TZ]. Msoka EF 2021 [40]*<br><br>*'…Maasai are living very far from the health centres because they tend to move from place to place far from town looking for the pasture for their animals…' [TB Care Giver,8 TZ]. Msoka EF 2021 [40]*
Individual	Poverty/ financial constraint, inability to pay the cost of treatment and other expenditures on travelling, food, accommodation, etc.	*"They have to stay in a hotel after coming here. They do not know that the sputum test including medication does not cost any money. However, they require money to travel from distant places and then they have to stay in a hotel. Sputum tests require a test for three consecutive days and thus diagnosis takes time and they have to bear the cost of waiting". (IDI, Health staff, Kaski) Babu Marahatta S 2020 [39]*<br><br>*"No money, no treatment - money plays a great role." (a woman (teacher, 47 years old) from Varzob town) Aye KW 2010 [38]*
Interpersonal	Stigma associated with TB/ cultural beliefs and practices, discrimination within society	*'…I used to be fearful; ashamed I could not move a lot because I knew people were talking about me. I stayed in the house. I would just get outside next to my house and warm myself in the sun because I did not have visitors, they did not want to come…' [TB patient, 7 KE]. Msoka EF 2021 [40]*<br><br>*"I was ashamed. I felt embarrassed because I had TB." (IDI1, 50yo, male, urban, short delay) Teo AKJ 2020 [41]*
Organisational	Inadequate infrastructure, limited access to TB diagnostic and treatment services	*People go to the hospital, they do not have a laboratory, they are indicated to go to other health units, but the patients do not have money to transport them. (MTS) Mitano F 2018 [42]*<br>*"Establishment of a health facility in a particular area is determined by population*

		density of that area. For instance, health post which is the lowest in the hierarchy, as a rule serves 3000-5000 people. As pastoralists are sparsely dispersed into large geographical area, they can hardly meet this condition" Abdi AG 2010 [26]
Organisational	Inadequate human resources and inadequate incentives to staff	*"Doctors in the health facility are not regular. So, people are examined by the health assistants and Auxiliary Nurse Midwife. . . we have the case that they provided wrong medicine due to which the patient's problems got complicated." Few years ago, one of my neighbour travelled to Pokhara and was found to have TB. This could easily have been done here" (FGD with six community members, Mustang, Lete) Babu Marahatta S 2020 [39]*<br><br>*"The health facilities in the rural areas are empty of staff because those areas are hard to reach due to insecurity combined with poor infrastructure. Accordingly, the health workers in the rural parts of the region may not receive salary, sometimes for several months. They often come back to Jigjiga [the capital town] and they never go back again". Abdi AG 2010 [26]*
Organisational	Distance to health facility, poor road connectivity, difficult terrain, hard to reach areas, seasonal difficulties like heavy rain	*'…so from here to our hospital is a bit far since we are in the interior and especially when we have rains like this time, to travel from here to the hospital is very difficult because accessibility of roads is very hard…' [Care giver, 6 TZ]. Msoka 2021 [40]*<br>*We know that in the Mozambican reality most of the inhabitants live, we speak of approximately 40 to 45% of inhabitants who live more than 8 km from the nearest health unit. (NPS6) Mitano F 2018 [42]*
Organisational	Lack of public transportation (accessibility)	*The main problem is transport as we get to the units when our access roads are damaged? (NPS5) Mitano F 2018 [42]*
Organisational	Symptomatic treatment by the health care providers	*The first time I was prescribed chloramphenicol tablets for 2 weeks. Since the hip pain remained, I went back to the hospital 2 weeks later. I was given amoxicillin for 2 months and a pelvic X-ray was taken. They told me I might be having osteal TB but I did not get TB treatment […] I went to the hospital for the third time. They told me to continue amoxicillin for 2 more weeks. (Laura, 61-year-old female, hip osteal TB) Verhagen LM 2010 [35]*
Public policy	Indirect cost/ Out of Pocket cost	*'…there are few times when I skipped my dose if I did not have enough food to eat, because if you take those drugs on an empty stomach, they make you feel very bad for like two three hours'. [TB Patient, 10 KE] Msoka EF 2021 [40]*<br><br>*"Some people in our community possess few goats. Such people can hardly seek TB*

		care because they cannot pay the cost" (Nomadic pastoralist, Jigjiga DOT Center). Abdi AG 2010 [26]
	**Theme: Enablers of TB Diagnosis**	**Quotes**
Interpersonal	Encouragement from family members, social support	*"My mother saw me coughing, and she said that I should go for [TB] screening." (IDI12, 33yo, male, long delay) Teo AKJ 2020 [41]*

DOT: Directly observed therapy; FGD: Focus group discussion; IDI: In-depth interview

Table S5: Interventions to improve TB diagnosis and notification

Study	Intervention	Intervention delivered by
Yassin MA 2013 [48]	1. Familiarization and awareness creation workshops<br>2. Health Extension Workers (HEW) training<br>3. Staff and laboratory technician training and microscope distribution<br>4. Advocacy, communication and social mobilization (ACSM) activities<br>5. HEWs identified people having cough for two or more weeks (Active case finding) (ACF)<br>6. Processing slides<br>7. Treatment initiation and screening household contacts<br>8. Treatment monitoring<br>9. Routine Recording and Reporting system<br>10. Feedback<br>11. Monitoring and Evaluation	The package was implemented in close collaboration with the Regional Health Bureau and the National TB Program (NTP).<br><br>TB team and Health Extension Workers (HEW)
Legesse Tesfaye YKL 2020 [47]	Household contact tracing of index TB cases	Healthcare workers
Shamnewadi AN 2020 [49]	Active Case Finding (ACF)	Healthcare workers in pre-identified high-risk areas.
Berhene Megerssa Ereso SAY 2020 [46]	TB Case Finding	1. PCF (Passive case finding) - delivered by healthcare providers at the health facility level.<br>2. Enhanced tb case finding- delivered by health extension workers (HEW) at the community level.<br>3. Intensified case finding- used for high-risk patients such as HIV infected individuals.<br>4. Active case finding
Der JB 2022 [45]	TB Case Finding	A TB team is made up of facility stakeholders

Table S6: Thematic representation of barriers and enablers of interventions, according to the CFIR framework

CFIR Framework	Theme: Barriers of interventions	References	Quotes
Intervention characteristics	Mismanagement of laboratory equipment used for diagnostic purposes.	2 (Der JB 2022, Berhene Megerssa Ereso SAY 2020) [45, 46]	➢ *"[...] sometime sputum container will get finish; cartridge will get finish and sometimes what marvels me is that on days containers are in the lab they will be giving the containers out without checking their stock levels but will wait when I send a patient to them for sputum test before they will tell me that containers are finished. Sometimes containers will get finish and because the clients are coming plenty and everybody is at risk, so I have to go and [...] have to give me some money to go and look for containers [...]."* (Male HCW, MH) Der JB 2022. [45]
	Limited or no diagnostic facilities, long distance between health centers leads to diagnostic delays.	2 (Der JB 2022, Berhene Megerssa Ereso SAY 2020) [45, 46]	➢ *"[. . .]. Our major problem is the absence of laboratory service in our health center, we are collecting sputum and sending it to other health centers for acid fast bacilli (AFB), and even we do not have a budget to do this." (A DOT provider)* Berhene Megerssa Ereso SAY 2020. [46]
	Target oriented approach of the field staff leading to the poor quality of active case finding.	1 (Shamanewadi AN 2020) [49]	➢ *"It is better to get samples of symptomatic patients. Sometimes the ASHA workers get samples of everyone to complete the target. And sometimes they get saliva instead of sputum to achieve their target. They bring inadequate quantity of sputum." (Said by a lab technician)* Shamanewadi AN 2020. [49]
Outer setting	Perceived traditional beliefs and stigma within the community.	3 (Der JB 2022, Shamanewadi AN 2020, Legesse Tesfaye YKL 2020) [45, 47, 49]	➢ *"Sometimes the level of stigmatization in the community is very high. Sometimes most of them they don't want their relatives and their loved ones and their friends to know that they have the condition so they may be hiding unless we are able to get them, talk to them and bring them in for treatment." (Male HCW, MH)* Der JB 2022. [45]
	Financial constraints of the patient, which impede access to healthcare services.	3 (Der JB 2022, Shamanewadi AN 2020, Berhene Megerssa Ereso SAY 2020) [45, 46, 49]	➢ *"[...] if they hear the name X (municipal hospital) they will start crying because they don't have money for T&T (travel and transportation)." (Female HCW, RHF).* Der JB 2022. [45]

	Lack of knowledge hinders testing of asymptomatic individuals during contact tracing.	2 (Legesse Tesfaye YKL 2020, Der JB 2022) [45, 47]	➢ *"We have not coughed or not become sickened. So, for what screening we go? If an individual doesn't have a cough, so he/she go for screening for TB?" (45year old, female, household contact of TB patient)* Legesse Tesfaye YKL 2020. [47]
	Disrespect towards field staff by community members.	1 (Shamanewadi AN 2020) [49]	➢ *"Many times, people don't cooperate. People get irritated if the ASHA workers go to their house. They just don't talk properly and try to send them away by saying that they don't have any problem." (As told by one TBHV)* Shamanewadi AN 2020. [49]
Inner setting	Low monetary incentives to government staff, impacting motivation and performance.	1 (Shamanewadi AN 2020) [49]	
	Lack of training on sputum collection and tuberculosis active case finding.	3 (Der JB 2022, Shamanewadi AN 2020, Legesse Tesfaye YKL 2020) [45, 47, 49]	➢ *"The main problem is sometimes they don't get proper quantity and most times they get saliva and put pressure on us to do the test and if I do test then if the sample is not good, we have to do smear, chemicals will be wasted, cups waste and our time will be wasted. So, ASHA workers have to be trained more about how to collect sputum." (LT)* Shamanewadi AN 2020. *[49]*
	Insufficient monitoring and supervision by higher authorities of the health care organisations.	2 (Der JB 2022, Legesse Tesfaye YKL 2020) [45, 47]	➢ *"There is no regular monitoring of household contact screening for index Tb cases. . ." (25-year-old, female, TB Focal)* Legesse Tesfaye YKL 2020. [47]
	Inadequate basic infrastructure and inappropriate design of health facility.	2 (Legesse Tesfaye YKL 2020, Berhene Megerssa Ereso SAY 2020) [46, 47]	➢ *"We are facing a great challenge from shortage of electricity power. Most of the health centers didn't have TB rooms and even the available ones are also not well ventilated. . . There is a shortage of reagents. . .& In addition to this, some microscopes do not make objects [bacilli] visible correctly." (29-year-old, male, TB Coordinator)* Legesse Tesfaye YKL 2020. [47]
	Waiting time and poor level of hygiene at the government hospitals.	2 (Legesse Tesfaye YKL 2020, Shamanewadi AN 2020) [47, 49]	➢ *". . .But there is a long waiting time at the health center due to a high number of clients. The past document was not found in the card room on time. Sometime the card would be sent to another service unit even if I wait in another room. Some health workers do not come on time or work in another class. I would wait some hours until they open the door of service." (30-year-old, male, TB patient)* Legesse Tesfaye YKL 2020. [47]
	Workload on health care professionals.	1 (Legesse Tesfaye YKL 2020) [47]	➢ *"TB focal has been working at another case team of health centers like Outpatient department, under-five clinics or emergency department besides of TB unit." (35 years old, female, HEW)* Legesse Tesfaye YKL 2020. [47]

	Closed training institutes.	1 (Legesse Tesfaye YKL 2020) [47]	
Individual characteristics	Gaps in TB knowledge and lack of training in case detection guidelines.	2 (Der JB 2022, Legesse Tesfaye YKL 2020) [45, 47]	➢ *"The nurses at the hospital here we are many and if all of us we know something about tuberculosis and about tuberculosis detection, if all of us we are aware and we know much about it, we will be able to identify more cases but because most of us don't know anything about tuberculosis when a person is even coughing, they will ignore the person until those experience ones capture the person." (Male HCW, MH)* Der JB 2022. [45]
	Healthcare workers' fear of TB infection and negative attitudes toward TB patients hinder effective early diagnosis.	2 (Der JB 2022, Berhene Megerssa Ereso SAY 2020) [45, 46]	➢ *"So, everybody is on the alert, I don't want to be infected, I don't want to get infected and that thing has brought in some reluctance in getting closer to TB clients or TB unit [...]." (Male HCW, MH)* Der JB 2022. [45]
	Poor counselling of patients by field staff.	1 (Shamanewadi AN 2020) [49]	➢ *"The problem is though we tell some people don't take proper treatment that is because we are lagging behind in giving them proper counselling. So patient counselling as well as family counselling is a very important part and as a supervisor, I feel that counselling component should be strengthened." (Said a TBHV)* Shamanewadi AN 2020. [49]
Implementation process	Lack of isolation facilities for coughing patients, compounded by the necessity to fast-track them, and creates confusion and frustration among both staff and other waiting patients.	1 (Der JB 2022) [45]	➢ *"You only tell them to use their handkerchief to cover their mouth when they are coughing because this place is just too small so you can't be isolating [...] and sometimes too some came to meet others so if they are overpassing it will bring confusion." (Female HCW, RHF)* Der JB 2022. [45]
	Low community mobilisation.<br>Inappropriate resource mobilisation.<br>Inadequate budget allocation and planning.<br>Low collaboration with other sectors<br>Inadequate expansion of health facilities to the community.	4 (Berhene Megerssa Ereso SAY 2020, Legesse Tesfaye YKL 2020, Der JB 2022, Shamanewadi AN 2020) [45, 46, 47, 49]	
	Complicated procedures of referral, feedback, and linkage to the health facility leads to the delayed diagnosis of TB.	1 (Legesse Tesfaye YKL 2020) [47]	➢ *"... But there was a gap in giving information of index case and feedback to health extension workers due to this they did not screen and refer household contacts to health center regularly." (25-year-old, female, TB Focal)* Legesse Tesfaye YKL 2020. [45]

	Ineffective management of field hours leading to an inability to interact with the community.	1 (Shamanewadi AN 2020) [49]	➢ *"Usually, we should go when people will be at home, but health workers, volunteers' don't follow time. If we tell them to go at 8 am, these staff delay and go by 9 am or 9.30 am. We tell them to either go in the morning or in the evening, then only it will be successful." (As told by one of the STS) "Almost all come for sputum examination, but they will see their convenience and we have to adjust to their timings. Few people give some reasons and don't turn up. And sometimes they escape by saying that they don't have interest, they have some important work." (Said a lab technician)* Shamanewadi AN 2020. [47]
**CFIR Framework**	**Theme: Enablers of intervention**	**References**	**Quotes**
Intervention characteristics	Provision of mobile phone airtime fees to HCWs and supervisors for communication.	1 (Yassin MA 2013) [48]	
	Provision of motorbikes for supervisors to transport smeared-slides and return test results and treatment.	1 (Yassin MA 2013) [48]	
	Use of light-emitting-diodes-fluorescent microscopes for laboratories.	1 (Yassin MA 2013) [48]	
	Advocacy, communication, and social mobilisation component delivering messages about TB and the availability of services during community meetings, campaigns, and local radios.	1 (Yassin MA 2013) [48]	
Outer setting	Positive attitude of community members to the intervention and creating awareness in the community	1 (Shamanewadi AN 2020) [49]	*"Really thankful to the ACF activity because of which I went for sputum testing and was diagnosed early and immediately started on treatment. Otherwise, I would have neglected and not got test done and probably my condition would have become complicated." (Said by male patient 3)* Shamanewadi AN 2020. [47]
	Engagement with stakeholders, including political, community, and religious leaders, teachers, and other stakeholders.	1 (Yassin MA 2013) [48]	

	Community meetings, campaigns, and local radio broadcasts to disseminate TB-related information.	1 (Yassin MA 2013) [48]	
Inner setting	Health workers familiarity with the work area where intervention is being implemented and the community recognising the field staff.	1 (Shamanewadi AN 2020) [49]	➤ *"ASHA workers don't face much problem because usually they will be allotted their area only for survey where people will be knowing them so they will not have much problem." (Said a laboratory technician)* Shamanewadi AN 2020. [47]
	Capacity strengthening workshops for the Health Bureau, HCWs, and laboratory staff.	1 (Yassin MA 2013) [48]	
	Familiarisation and awareness creation workshops for political, community, and religious leaders, teachers, and other stakeholders.	1 (Yassin MA 2013) [48]	
	Appointment of one project supervisor per district to support and supervise field activities.	1 (Yassin MA 2013) [48]	
Individual characteristics	Perceived usefulness of active case finding among the field staff as it creates awareness among community and is useful to detect tuberculosis among the high-risk groups.	2 (Shamanewadi AN 2020, Berhene Megerssa Ereso SAY 2020) [46, 49]	➤ *"With this survey, most importantly awareness is being created among the people in the community. In this ACF, high risk groups are found out and because of this, high - risk group people are motivated to come for sputum testing. People think that from health department people are coming to tell us and they feel good and they get the test done." (Said a STS)* Berhene Megerssa Ereso SAY 2020. [46]
	Training workshops for Health Bureau, HCWs, and laboratory staff to enhance individual skills and knowledge.	1 (Yassin MA 2013) [48]	
Implementation process	Implementation of tailored training workshops.	1 (Yassin MA 2013) [48]	
	Supervision of field activities by appointed project supervisors	1 (Yassin MA 2013) [48]	

ACF: Active case finding; ASHA: Accredited social health activist; DOTS: Directly observed therapy short course; HCW: Health care worker; MH: Municipal hospital; RHF: Rural health facility; STS: Senior Treatment Supervisor; TBHV: TB health visitor
